# The association between Bacillus Calmette-Guérin vaccination (1331 SSI) skin reaction and subsequent scar development in infants

**DOI:** 10.1186/s12879-017-2641-0

**Published:** 2017-08-03

**Authors:** Nina Marie Birk, Thomas Nørrelykke Nissen, Monica Ladekarl, Vera Zingmark, Jesper Kjærgaard, Trine Mølbæk Jensen, Signe Kjeldgaard Jensen, Lisbeth Marianne Thøstesen, Poul-Erik Kofoed, Lone Graff Stensballe, Andreas Andersen, Ole Pryds, Susanne Dam Nielsen, Christine Stabell Benn, Dorthe Lisbeth Jeppesen

**Affiliations:** 1 Department of Pediatrics, Copenhagen University Hospital, Hvidovre, Denmark; 2 The Department of Pediatrics and Adolescent Medicine, Juliane Marie Centret, Rigshospitalet, Copenhagen University Hospital, Copenhagen, Denmark; 3 Department of Pediatrics, Kolding Hospital, Kolding, Denmark; 4 Research Center for Vitamins and Vaccines (CVIVA), Bandim Health Project, Statens Serum Institut, Copenhagen, Denmark; 5 Department of Infectious Diseases, Rigshospitalet, Copenhagen University Hospital, Copenhagen, Denmark

## Abstract

**Background:**

The Bacillus Calmette-Guérin vaccine (BCG) against tuberculosis is administered intradermally, and vaccination is often followed by a scar at the injection site. Among BCG-vaccinated individuals, having a scar has been associated with lower mortality. We aimed to examine the impact of vaccination technique for scarring in a high income setting, by assessing the associations between the post injection reaction, the wheal size, and the probability of developing a scar, and scar size.

**Methods:**

This study was nested within a clinical multicenter study randomizing 4262 infants to either BCG vaccination (BCG 1331 SSI) or no intervention. In this substudy, including 492 vaccinated infants, the immediate post BCG vaccination reaction was registered as either wheal (a raised, blanched papule at the injection site), bulge (a palpable element at the injection site), or no reaction. The presence or absence of a BCG scar and the size the scar was measured at 13 months of age.

**Results:**

Of 492 infants included, 87% had a wheal after vaccination, 11% had a bulge, and 2% had no reaction. The mean wheal size was 3.8 mm (95% confidence interval 3.7–3.9). Overall, 95% (442/466, 26 lost to follow-up) of BCG-vaccinated infants had a scar at 13 months of age. In infants with a wheal, the probability of developing a scar was 96%, declining to 87% in the case of a bulge, and to 56% in the case of no reaction (p for same probability = 0.03). Wheal size was positively correlated with the probability of getting a scar and scar size.

**Conclusion:**

Scarring after BCG vaccination has been associated with lower infant mortality. In a high-income setting, we found that correct injection technique is highly important for the development of a BCG scar and that registration of the category of BCG skin reaction (as wheal, bulge, or no reaction) may be used to identify infants at risk of scar failure. Finally, the wheal size was positively associated with both the probability of getting a scar and scar size.

**Trial registration:**

The study was registered at www.ClinicalTrials.gov with trial registration number NCT01694108.

**Electronic supplementary material:**

The online version of this article (doi:10.1186/s12879-017-2641-0) contains supplementary material, which is available to authorized users.

## Background

Tuberculosis (TB) still represents a great threat to global health. It is estimated that approximately one third of the world’s population is currently infected with latent TB, and 2 million people die of TB each year [1]. Despite ongoing efforts to develop new and more effective TB vaccines [2], BCG remains the only licensed vaccine against TB. BCG has been administered to billions of infants and is one of the most commonly used vaccines worldwide [3].

Following a correct administered intradermal BCG vaccination, a transient post injection wheal is usually observed; often, but not always, followed by a pustule and a flat scar at the injection site [4]. The presence or absence of a scar is often used as an indicator of BCG vaccination in a clinical context as well as in health surveys to assess vaccine coverage [5]. However, not all children develop a scar. Several factors, such as correct vaccination technique [3, 6], dose of vaccine [7, 8], BCG strain [9–11], sex of the child [6, 12], age at vaccination [12–14], and prior tuberculin sensitivity [15] have been described to impact scarring. Furthermore, immune characteristics in the infant prior to vaccination may be important for scar development [10]. Importantly, among BCG-vaccinated individuals, having a scar has been associated with reduced mortality [16–18] and in a recent study conducted in West Africa, mortality for scar-positive infants was found to be half that of scar-negative infants [10].

Between 2012 and 2015, we conducted The Danish Calmette Study [19], a randomized multicenter trial aiming to study the impact of neonatal BCG on morbidity outcomes. Despite the widespread use of BCG through decades there is no standard or point of reference for the measurement of post vaccination reaction or scar assessment. In the present substudy, we aimed to examine the importance of vaccination technique for scarring using the BCG (1331 SSI) in a setting with no routine BCG vaccination and a low prevalence of TB [20] and HIV [21], providing reference material for measurements of post vaccination reaction and scar size and frequency in a high income setting. We assessed the association between the post injection reaction, the wheal size and the probability of developing a scar and scar size. We hypothesized that having a wheal would be associated with a higher scar frequency, and that an increase in wheal size would increase the probability of getting a scar and the size of the scar.

## Methods

### Setting

This substudy was nested within the Danish Calmette study, described in detail elsewhere [19]. In brief, infants were randomized within 7 days of birth with a 1:1 allocation to either BCG vaccination or no intervention. The inclusion criteria were gestational age ≥ 32 weeks and a birth weight ≥ 1000 g. Exclusion criteria were maternal intake of immune modulating medicine during pregnancy or signs of severe illness or major malformation in the newborn. A total of 4262 infants were enrolled, and children who were allocated to BCG vaccination received the BCG strain SSI 1331. The vaccination procedure was conducted according to the manufacturer’s instructions [22], using the standard-of-care intradermal Mantoux injection technique [23]. The vaccine was stored at 2–8 degrees °C at the maternity wards prior to use. A sterile 1 mL syringe with a short (25–27 G) needle was used for the injection of 0.05 mL of the vaccine suspension intradermally in the deltoid region of the left arm.

### The present study

**Fig. 1:**
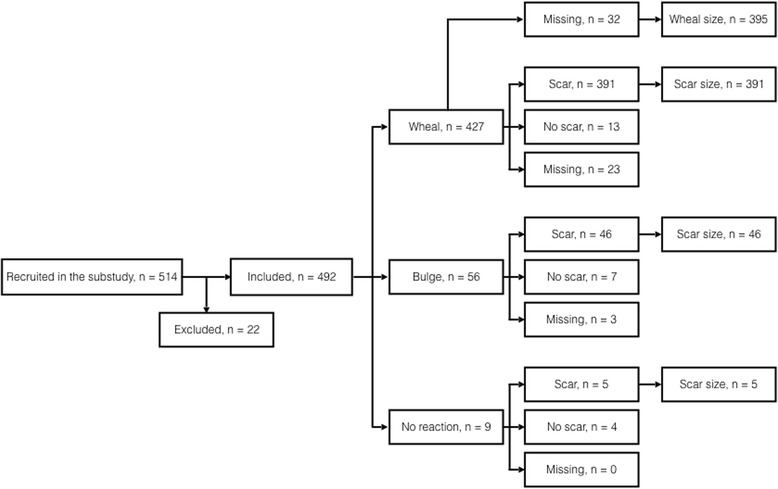
Inclusion into the study on the association between BCG vaccination skin reaction (categorized as wheal, bulge, or no reaction) and the subsequent development of a scar

The present substudy was conducted at two of the three randomization sites: Copenhagen University Hospital, Hvidovre and Copenhagen University Hospital, Rigshospitalet. From February 7th to November 28th 2013, infants randomized to BCG by two members of our study staff (A and B) at Copenhagen University Hospital, Hvidovre, were included into this substudy. From May 29th to October 31st 2013 infants randomized to BCG by one member of our study staff (C) at Copenhagen University Hospital, Rigshospitalet, were included. We aimed to include a minimum of 500 infants (Fig. 1).

### Measurements of the BCG vaccination skin reaction and scar

**Fig. 2:**
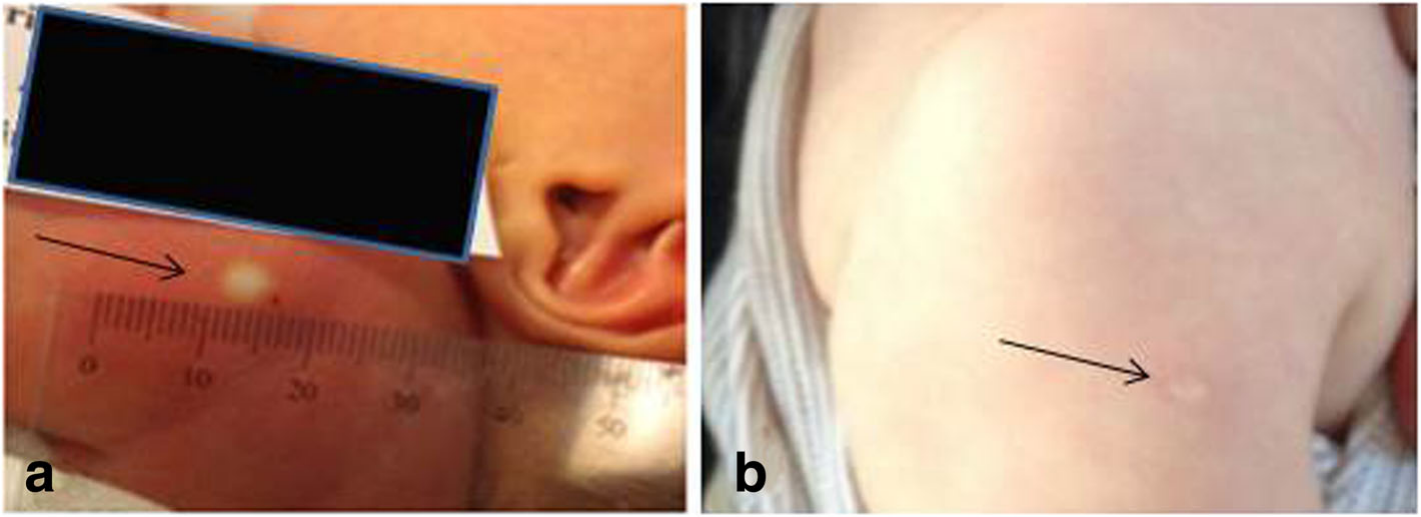
The association between BCG vaccination skin reaction and the subsequent development of a scar. **a** Measurement of the size of a BCG vaccination wheal at birth. **b** Subsequent round and flat scar on the infant’s upper left arm at 13 months of age

Immediately after the administration of the BCG vaccine, the size of the BCG vaccination skin reaction was assessed. Firstly, the skin reaction was categorized as either A) “wheal”, if a raised, blanched papule was visible at the injection site (Fig. 2a), B) “bulge”, if no visible wheal emerged but there was a palpable bulge, or C) no reaction [6]. Secondly, in the case of a wheal, the longest diameter or length (as not all wheals were round in shape) of the wheal was measured (mm) once with a transparent ruler (Tuberculin PPD RT 23 SSI, Copenhagen, Denmark) (Fig. 2a). The assessors of the BCG vaccination skin reaction were midwifes (A and C), and a medical student (B) trained in the procedure of BCG vaccination and measurement of BCG vaccination skin reaction in connection with the present study. None of the assessors were experienced prior to commencement of the study.

The presence of a scar (yes/no) and the scar size (mm) was assessed within the entire Danish Calmette Study at the age of 13 months (Fig. 2b). The longest diameter or length (as not all scars were round in shape) of the scar was measured twice, followed by a repeated measurement of the diameter/length perpendicular to this. The mean of these four measurements was used as the estimate of scar size.

### Statistical methods

The association between BCG vaccination skin reaction (wheal, bulge, no reaction) and scar (yes/no) as well as the association between wheal size (mm) and scar (yes/no) were analyzed as prevalence data assessed in Poisson regression models with robust variance estimates [24], with the scar variables as outcomes and the BCG vaccination skin reaction category and wheal size, respectively, as covariates. Among children with a scar, the association between the size of the wheal (mm) and the size of the scar (mm) was examined using linear regression analysis, with the scar size as outcome and wheal size as covariate. One scar size estimate of 27.5 mm was considered an outlier and was excluded from the analysis.

On the basis of previous findings that the ability of BCG to induce scars may be differentiated by sex [10] both overall and sex-stratified estimates were calculated.

Sex of the child, vaccinator (A, B, C), and study months (months 1–12, since season of birth is thought to affect the immune system) were assessed as potential confounders by including them as covariates in the statistical models. Since birth weight was positively associated with wheal size (but not with category of BCG skin reaction, scar size, or scar frequency), birth weight was also included as a covariate in the statistical analyses concerning wheal size.

Analysis of predefined potential effect modifiers: sex of the child, vaccinator (A, B, C), age at vaccination (0–1 day vs. 2–7 days), season, study months (1–12), scar examiner (1–9), and maternal BCG (yes/no) were conducted. A 5%-significance level was used. All analyses were performed using STATA 13.1 (StataCorp LP, College Station, Texas).

### Ethical considerations

This substudy, as part of the protocol of the main trial, was approved by the Committees on Biomedical Research Ethics (J.no. H-3-2010-087), the Danish Data Protection Board (J.no. 2009–41-4141), and the Danish Medicines Agency (J.no.2612–4356.EudraCT 2010–021979-85. Protocol 2009–323). The study was registered at www.ClinicalTrials.gov with trial registration number NCT01694108. The trial was supervised by the Good Clinical Practice Units of the Capital Region, Denmark and monitored by an independent Data Safety Monitoring board. This study was performed in accordance with the Declaration of Helsinki. Infants were included after both oral and written information had been provided and a signed consent form from both parents had been obtained.

## Results

A total of 514 BCG-vaccinated infants were eligible for inclusion into this substudy; 22 were excluded due to lack of registration that enabled pairing of outcome and child in the case of twins, or erroneously assigned study id numbers on the sheet where the BCG skin reaction was registered (Fig. 1). Of the remaining 492 infants, 87% (427/492) had a wheal after vaccination, 11% (56/492) had a bulge, and 2% (9/492) had no reaction. Overall, 95% (442/466, 26 lost to follow-up) of BCG-vaccinated infants developed a scar, in equal number of boys and girls (Fig. 1).

Baseline characteristics for infants in this study are shown in Table 1. Baseline characteristics in the present study were similar to those of infants in The Danish Calmette Study, except for ‘age at vaccination’; 15% of infants in this study were vaccinated <1 day of age vs. 48% in the Danish Calmette study, *p* < 0.001 (Additional file 1: Table S1A). This was due to the fact that Copenhagen University Hospital, Hvidovre generally included children 1–2 days later than the two other recruitment sites.

**Table 1:** Baseline characteristics for children randomized to BCG in the study on the association between BCG vaccination skin reaction and the development of a scar

	BCG skin reaction & scar study
	*n* = 492
Sex (male)^a^	257 (52%)
Prematurity (GA < 37)	10 (2%)
Caesarean section	108 (22%)
Birth weight in grams (mean ± SD)	3501 ± 504
Age at time of randomization <1 day	75 (15%)
Maternal BCG	92 (19%) [2]
At least one parent of non-Danish ethnicity	98 (20%)[0]
Maternal smoking during pregnancy	49 (10%)[0]
Level of maternal education	[1]
*Basic schooling and non-theoretical education*	96 (20%)
*Theoretical education incl. BA level*	212 (43%)
*Master level or more*	183 (37%)
Siblings	192 (39%)
Atopic predisposition^b^	336 (68%)

^a^ n number (Frequency) [not available] unless otherwise stated

^b^ Atopic predisposition defined as at least one first degree relative with atopic disease. Atopic disease is defined as physician-diagnosed atopic eczema, asthma, and allergic rhino conjunctivitis or food allergy

### BCG vaccination wheal size, scar size, and scar rate

In infants with a wheal, the mean wheal size was 3.8 mm, 95% confidence interval (CI) (3.7–3.9); 3.9 mm for boys and 3.8 mm for girls (*p* = 0.09 for same wheal size in boys and girls). The overall mean scar size was 4.6 mm, 95% CI (4.5–4.8), 4.7 mm for boys and 4.5 mm for girls (*p* = 0.06 for same scar size in boys and girls) (Table 2). The three vaccinators did not produce same wheal size (range 3.5–4.3, *p* < 0.001), or scar frequency (range 93–100%, *p* < 0.001) (Table 2). Baseline characteristics for infants vaccinated by the three vaccinators differed for level of maternal education, *p* = 0.002 (Additional file 2: Table S2B).

**Table 2:** An overview of the outcomes of the study assessing BCG vaccination skin reaction and subsequent scaring - stratified by sex and vaccinator

	Wheal		Bulge		No reaction		Wheal size (mm)		Scar		No scar		Scar size (mm)	
	n	(%)	n	(%)	n	(%)	mean (95% CI)	n	n	(%)	n	(%)	mean (95% CI)	n
Total	427	(87)	56	(11)	9	(2)	3.8 (3.7–3.9)	395	442	(95)	24	(5)	4.6 (4.5–4.8)	442
Boy	227	(88)	29	(11)	1^a^	(1)	3.9 (3.7–3.9)	205	232	(95)	12	(5)	4.7 (4.5–4.8)	232
Girl	200	(85)	27	(12)	8	(3)	3.8 (3.6–3.9)	190	210	(95)	12	(5)	4.5 (4.3–4.7)	210
Vaccinator														
A	307	(84)	49	(14)	8	(2)	3.8 (3.7–3.9)	280	326	(94)	20	(5)	4.7 (4.5–4.9)	326
B	59	(92)	4	(6)	1	(2)	3.5 (3.3–3.8)	55	53	(93)	4	(6)	4.1 (3.8–4.5)	53
C	61	(95)	3	(5)	0	(0)	4.3 (4.1–4.4)^b^	60	63	(100)^c^	0	(0)	4.8 (4.5–5.1)	63

^a^The categories of “wheal”, “bulge”, and “no reaction” were unevenly distributed between sexes, *p* = 0.04

^b^There was a significant difference in wheal size between the three vaccinators, *p* < 0.001

^c^There was a significant difference in scar rate between the three vaccinators, *p* < 0.001

### The association between category of BCG vaccination skin reaction (wheal/bulge/no reaction) and scar

**Fig. 3:**
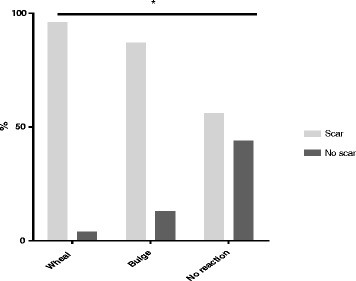
The association between three categories of BCG vaccination skin reaction (wheal, bulge, or no reaction) registered immediately after neonatal BCG vaccination and the probability (%) of developing a scar at 13 months of age. The IRR of getting a scar preceded by a ‘bulge’ (with ´wheal´ as reference), was 0.90; the IRR of getting a scar preceded by ´ no reaction ´ was 0.57, *indicates *p* = 0.03

The probability of developing a scar following a post vaccination wheal was 96%. The probability of developing a scar was 87% following a post vaccination bulge and 56% following no visible or palpable post vaccination reaction (Fig. 3). The incidence rate ratio (IRR) of getting a scar preceded by a bulge was 0.90, 95% CI (0.80–0.98) (wheal as reference). Furthermore, the IRR of getting a scar preceded by no reaction was 0.57, 95% CI (0.31–0.99) (wheal as reference). The difference between the three types of BCG skin reactions and the subsequent probability of developing a scar was significant (*p* = 0.03). Adjustment for sex of the child, vaccinator, and study months (1–12) did not change the result: the aIRR of getting a scar preceded by a bulge was 0.90; the aIRR of getting a scar preceded by no reaction was 0.58, (*p* < 0.001).

The association between BCG vaccination skin reaction and BCG scar was different for the three vaccinators (p for same association = 0.008): Vaccinator A had an IRR for bulge of 0.87, 95% CI (0.77–0.99) and an IRR of 0.52, 95% CI (0.26–1.03) for no reaction. Vaccinator B had an IRR for bulge of 1.08, 95% CI (1.01–1.20), and IRR = 1.08, 95% CI (1.03–1.15) for no reaction. Vaccinator C could not be evaluated, as there were no children with no reaction.

The association between BCG vaccination skin reaction and BCG scar was similar across age at vaccination, sex, season, study months, maternal BCG, and scar examiner (data not shown).

### The association between size of BCG vaccination wheal and the development of a scar

In infants with a BCG vaccination wheal, we found an association between wheal size and the prevalence of having a scar, the birth weight adjusted aIRR being 1.03, 95% CI (1.01–1.06), *p* = 0.005. This corresponds to a 3% increase in the probability of getting a scar, when wheal size increased by 1 mm. Adjusting the analysis for sex, vaccinator, and study months did not change the result: IRR 1.03, 95% CI (1.01–1.05), *p* < 0.001.

Again, vaccinator was a significant effect modifier, the IRR in the association between wheal size and the prevalence of having a scar for vaccinator A was 1.04, 95% CI (1.01–1.07) and for B it was 0.99, 95% CI (0.96–1.02). Vaccinator C produced 100% scars, *p* value for interaction = 0.02.

The association between BCG vaccination wheal size and chance of a BCG scar was similar across age at vaccination, sex, season, study months, maternal BCG, and scar examiner (data not shown).

### BCG vaccination wheal size and scar size, among infants with a BCG scar

**Fig. 4:**
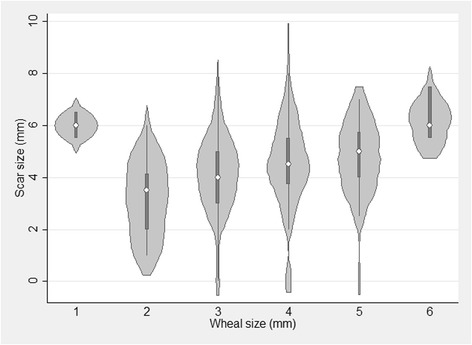
Violin plot of the association between size of the wheal and size of the scar measured at 13 months in infants vaccinated with BCG at birth. The open circle in the center of the plots depicts the median scar size at a given wheal size, the box depict the interquartile range, and the spikes depict the upper and lower values. The light gray areas reflect the density of the data

Assessing the association between the wheal and scar size by linear regression analyses, the birth weight adjusted regression coefficient was 0.10 mm/mm, 95% CI 0.01–0.19, *p* = 0.02. Hence, scar size increased with 0.1 mm when wheal size increased with 1 mm (Fig. 4).

After adjustment for sex, vaccinator, and study month, the regression coefficient was 0.11 mm/mm, 95% CI (0.03–0.21), *p* = 0.01.

The association between BCG vaccination wheal size and scar size was similar across vaccinators, age at vaccination, sex, season, study months, maternal BCG, and scar examiner (data not shown).

## Discussion

In a high-income setting, we found that the categories of post-vaccination skin reaction (wheal, bulge, or no reaction) predicted substantially different scar frequencies. Furthermore, an increase in wheal size raised both the probability of getting a scar and increase in scar size.

Previous reports of wheal size after the injection of 0.05 ml BCG intradermally vary from 2.0 mm (IQR 0.0–3.0) in newborns vaccinated within 48 h after birth [25] to 4.9 mm (mean) at 6 months of age [7] and 6.3 mm in school children [8]. Age has been shown to be positively associated with wheal size [7], and our mean wheal size (3.8 mm) may reflect the relatively early age of vaccination in our study (median age in days 1, 10–90 percentiles (1–2)) . However, infants in our study may be slightly older than the South African newborns (with a median wheal of 2.0 mm [25]) as infants were vaccinated within 7 days after birth in our study vs. within 48 h after birth in the study by Geldenheys et al. [25]. Differences in wheal size may also be due to genetic differences between the study populations or differences in exposure to environmental mycobacterial antigens [26]. Our overall scar frequency (95%) corresponds to scar frequencies found in studies from urban sub Saharan Africa (91–94%)^4,6,9^ and the UK (100%) [26].

The development of a scar after BCG vaccination has been used as one of the indicators for successful vaccination and in estimating vaccine coverage [12]. Since BCG is administered throughout the world as the only licensed preventive vaccine against TB, post-vaccination scaring as a BCG signature remains important. Although the efficacy of BCG in the protection against TB has been highly debated, a recent study from Greenland found BCG vaccination effective in reducing both mycobacterial infection and TB disease in children and adolescents [27]. The presence of a scar has not been found to correlate with the protection against TB [28, 29]; this may however reflect the varying efficacy of BCG against TB [30]. In a setting of routine BCG vaccination, studies from West Africa have shown that BCG vaccination may be associated with lower mortality [31, 32]. Among BCG-vaccinated individuals, having a scar was associated with further reduced mortality [16–18]. For instance, in a recent study from rural Guinea-Bissau, mortality for scar-positive infants was found to be half that of scar-negative infants [10]. This may be related to underlying differences in the immune systems of children who developed or did not develop a BCG scar. However, the association between having a scar and lower mortality was comparable in populations with low (52%) [10] and high(>90%) [16, 18]scar frequencies. Hence, it seems more likely that scar is a marker of a correctly administered BCG vaccine. These findings suggest that it may be important to monitor the development of a BCG scar as a marker of a well-functioning vaccination program. Future clinical trials may further examine if revaccinating infants with scar failure may improve survival in low-income countries.

Thus, confirming the findings from low-income settings, our results show that vaccination technique, i.e. correct intradermal administration of neonatal BCG is highly important for scarring. In our high-income setting, using the BCG SSI 1331, getting a wheal converts into a scar frequency of 93–100%, depending on vaccinator. Infants who developed a bulge, but no wheal, presumably got the vaccine subcutaneously [6]. This resulted in a lower scar frequency than administration leaving a wheal, which was visibly intradermal. These findings corroborate previous studies showing that subcutaneous administration of BCG induced less recognizable/fewer scars than intradermal administration [7] and that intradermal administration formed a higher scar rate (94%) compared to subcutaneous administration (82%) [6]; scar rates which were comparable to ours of 96% following intradermal BCG vs. 87% following subcutaneous BCG*.*

A marked reduction in scar frequency (56%) was seen when no reaction was formed post vaccination. This category, most likely covered both deep (intra cutaneous/intra muscular) administration of vaccine and no or little vaccine given (perforation of the skin or reflux of the vaccine through the injection canal). In a recent study from rural Guinea-Bissau, a surprisingly low overall scar frequency of 52% was found [10], comparable to our findings after ‘no reaction’. The authors suggest several explanatory factors, among them poor vaccination technique, due to less experienced and lack of specialized nurses in the rural areas. It was also suggested that BCG strain may play a role, since most of the children got the Russian BCG strain, which has been associated with a lower scar frequency in more studies [10, 33].

We also found that BCG vaccine wheal was directly associated with the size of the scar (Fig. 4). This again corroborates previous studies [7, 8], however, worth noting, the effect on scar size in the present study (regression coefficient = 0.10 mm/mm) indicates that determinants other than wheal size are important for scar size. Furthermore, as seen for scar frequency, wheal size and scar rate varied among vaccinators. These differences should, however, be seen in the light of the different inclusion periods into this study. Vaccinator C, who presented bigger wheals and scar rate, began enrollment close to 4 months after vaccinators A and B, and had therefore acquired more experience. When comparing baseline characteristics for infants vaccinated by the three vaccinators, only level of maternal education differed; suggesting that differences in baseline characteristics are not likely to explain the differences in wheal size and scar rate observed between vaccinators.

Despite the widespread use of BCG there is no gold standard for the measurement of post vaccination wheal or scar. This study reduces this knowledge gap by presenting reference material from a prospective study on healthy infants assessed within a standardized setup in a high income country with a low burden of TB and provides a unique possibility for further follow-up through the national registers.

A strength of this study is the standardized study setup; BCG vaccination was done by members of our study staff specifically trained in the vaccination procedure and in measuring outcomes according to the study protocol. A limitation lies within the different inclusion periods at the two sites, and experience level at inclusion start may have differed. Although the study included 492 infants, only 9 infants had no reaction post vaccination, and this is a limitation to the study. Mothers included in the Danish Calmette Study and hence in this substudy, were of higher education and parents had a higher prevalence of atopic predisposition than the background population [34] which may limit the generalizability to the overall Danish population. Of interest, but out of scope for this study, would have been to assess tuberculin skin test (TST) reaction, since studies have shown vaccination technique, dose of vaccine injected, and type of vaccine to determine both size and frequency of TST reaction as well as BCG scar [6, 18].

## Conclusion

In conclusion, we demonstrated that the skin reaction at the injection site is highly important for the probability of developing a scar after neonatal BCG vaccination (1331 SSI) in a high-income setting. Compared with presenting a wheal at the site of injection after BCG vaccination, having no reaction was associated with a reduced probability of developing a scar by >40%. Hence, training in correct injection technique may greatly improve the probability of getting a BCG scar. Registration of the category of BCG skin reaction immediately after vaccination may be used to identify infants at risk of scar failure. Focus on improving BCG vaccination technique may improve BCG scar frequency, with potential benefits for both vaccine coverage surveillance and child mortality in low-income countries.

## Additional files

Additional file 1: Table S1A. Comparison of baseline characteristics for BCG vaccinated children in the present substudy with BCG vaccinated children in the Danish Calmette Study. (DOCX 16 kb)

Additional file 2: Table S2B. Comparison of baseline characteristics between infants vaccinated by the three different vaccinators A, B and C. (DOCX 17 kb)

## Abbreviations

aIRR: adjusted incidence rate ratio
BCG: Calmette-Guérin vaccine
CI: Confidence interval
TB: Tuberculosis
