# Supplementary material for: The association between Bacillus Calmette-Guérin vaccination (1331 SSI) skin reaction and subsequent scar development in infants
Source: BMC Infect Dis. 2017 Aug 3;17:540. doi: 10.1186/s12879-017-2641-0 (PMC5541744; doi:10.1186/s12879-017-2641-0)
Supplement: Supplementary file 1 — Comparison of baseline characteristics for BCG vaccinated children in the present substudy with BCG vaccinated children in the Danish Calmette Study. (DOCX 16 kb) [file 12879_2017_2641_MOESM1_ESM.docx]

**Supplementary table A**. Comparison of baseline characteristics for BCG vaccinated children in the present substudy with BCG vaccinated children in the Danish Calmette Study

|  | **BCG skin reaction & scar substudy** | **Danish Calmette Study** | p value |
| --- | --- | --- | --- |
|  |  |  |  |
| **BCG vaccinated** | **n=492** | **n=2095^a^** |  |
| Sex (male)^b^ | 257 (52%) | 1092 (52%) | 1.0 |
| Prematurity (GA<37) | 10 (2%) | 61 (2.9%) | 0.3 |
| Caesarean section | 108 (22%) | 411 (20%) | 0.4 |
| Birth weight in grams (mean±SD) | 3501±504 | 3519±493 | 0.5 |
| Age at time of randomization < 1 day | 75 (15%) | 1006 (48%) | <0.001* |
| Maternal BCG | 92 (19%) [2] | 364 (18%) [27] | 0.6 |
| At least one parent of non-Danish ethnicity | 98 (20%)[0] | 376 (18%)[14] | 0.4 |
| Maternal smoking during pregnancy | 49 (10%)[0] | 203 (10%)[1] | 0.9 |
| Level of maternal education | [1] | [7] | 0.4 |
| *Basic schooling and non-theoretical education* | 96 (20%) | 460 (22%) |  |
| *Theoretical education incl. BA level* | 212 (43%) | 935 (45%) |  |
| *Master level or more* | 183 (37%) | 693 (33%) |  |
| Siblings | 192 (39%) | 887 (42%) [1] | 0.4 |
| Atopic predisposition^c^ | 336 (68%) | 1540 (74%) | 0.5 |
|  |  |  |  |
|  |  |  |  |
| ^a^ Mothers of infants allocated to BCG in The Danish Calmette Study | |  |  |
| ^b^ n number (Frequency) [not available] unless otherwise stated | |  |  |

^c^ Atopic predisposition defined as at least one first degree relative with atopic disease. Atopic disease is defined as physician-diagnosed atopic eczema, asthma, allergic rhinoconjunctivitis or food allergy.

* P < 0.05
