# Supplementary material for: The association between Bacillus Calmette-Guérin vaccination (1331 SSI) skin reaction and subsequent scar development in infants
Source: BMC Infect Dis. 2017 Aug 3;17:540. doi: 10.1186/s12879-017-2641-0 (PMC5541744; doi:10.1186/s12879-017-2641-0)
Supplement: Supplementary file 2 — Comparison of baseline characteristics between infants vaccinated by the three different vaccinators A, B and C. (DOCX 17 kb) [file 12879_2017_2641_MOESM2_ESM.docx]

|  | **Table 1**. Comparison of baseline characteristics for children randomized to BCG in the post-BCG vaccination Papule and Scar Study between the three vaccinators A, B, and C. | | | | | |
| --- | --- | --- | --- | --- | --- | --- |
|  |  | **Vaccinator A** | **Vaccinator B** | **Vaccinator C** | | p value |
|  |  |  |  |  | |  |
|  | **BCG vaccinated** | **n=364** | **n=64** | **n=64** | |  |
|  | Sex (male)^a^ | 190 (52%) | 36 (56%) | 31 (48%) | | 0.7 |
|  | Prematurity (GA<37) | 8 (2%) | 0 (0%) | 2 (2%) | | 0.4 |
|  | Caesarean section | 76 (21%) | 14 (22%) | 18 (28%) | | 0.6 |
|  | Birth weight in grams (mean±SD) | 3508±500 | 3502±439 | 3467±597 | | 0.9 |
|  | Age at time of randomization < 1 day | 53 (15%) | 11 (18%) | 11 (18%) | | 0.8 |
|  | Maternal BCG | 64 (18%) | 16 (25%) | 12 (19%) | | 0.3 |
|  | At least one parent of non-Danish etnicity | 73 (20%) | 8 (13%) | 17 (26%) | | 0.1 |
|  | Maternal smoking during pregnancy | 40 (11%) | 5 (9%) | 4 (6%) | | 0.5 |
|  | Level of maternal education |  |  |  | | **0.002** |
|  | *Basic schooling and non-theoretical education* | 75 (21%) | 15 (23%) | 6 (10%) | |  |
|  | *Theoretical education incl. BA level* | 163 (45%) | 30 (47%) | 20 (31%) | |  |
|  | *Master level or more* | 126 (34%) | 19 (30%) | 38 (59%) | |  |
|  | Siblings | 138 (38%) | 26 (41%) | 28 (43%) | | 0.9 |
|  | Atopic predisposition^b^ | 253 (70%) | 44 (69%) | 39 (61%) | | 0.4 |
|  |  |  |  |  | |  |
|  |  |  |  |  | |  |
|  | ^a^ n number (Frequency) unless otherwise stated | |  |  | |  |
|  | ^b^ Atopic predisposition defined as at least one first degree relavtive with atopic disease. Atopic disease is defined as physician-diagnosed atopic eczema, asthma, allergic rhino conjunctivitis or food allergy. | | |  |  |  |
|  |  | | |  |  |  |
|  |  |  |  |  |  |  |

**Supplementary Table B**
